# VEGF-C as a putative therapeutic target in cancer

**DOI:** 10.18632/oncotarget.27007

**Published:** 2019-06-18

**Authors:** Signe R. Michaelsen, Hans S. Poulsen, Petra Hamerlik

**Affiliations:** Copenhagen University Hospital, Copenhagen, Denmark; Danish Cancer Society, Copenhagen, Denmark

**Keywords:** VEGF-C, VEGF-A, VEGFR2, bevacizumab, glioblastoma, cancer

Members of the Vascular Endothelial Growth Factor (VEGF) pathway have been extensively studied as cancer targets. While originally acknowledged for their role in regulating the formation of blood and lymphatic vessels, recent studies implicated these factors in the regulation of additional processes crucial for carcinogenesis. For instance, we have previously reported the essential role for autocrine VEGF-A/VEGF Receptor 2 (VEGFR2) signaling in the survival and tumorigenic potential of glioblastoma (GBM) cells [1].

While the number of reports furthering our understanding of VEGF-A-mediated signaling in cancer is fairly high, only limited attention has been paid to the contribution of VEGF-C signaling in cancer. Recent reports mostly focuse on VEGF-C in regards to its role in the process of lymphangiogenesis, mediated primarily by a paracrine stimulation of VEGFR3 in endothelial cells [2]. While the expression of VEGF-C in adult healthy tissue is low [2], VEGF-C was found aberrantly expressed in tumor cells and tumor associated macrophages, where the over-expression informed adverse patient prognosis [3]. Eventhough VEGF-C over-expression has been reported for GBM [4], mechanistic insights into its function in GBM cells remained elusive. Our recent study, for the first time, sheded light on the molecular underpinnings of VEGF-C-mediated signaling in GBM cells [5]. Using a microscopy-based approach coupled with proximity ligation assay, we confirmed a direct interaction of VEGF-C and VEGFR2 in GBM tissue sections and a number of primary cell lines, indicating that besides its paracrine function, VEGF-C may operate in a an autocrine manner in VEGFR-expressing cancer cells. Our data show that VEGF-C/VEGFR2 signaling drives the survival and tumorigenicity of GBM cells. In particular, VEGF-C silencing reduced cell proliferation, activated cell cycle checkpoint, leading to cell cycle arrest and the induction of apoptosis. Our data functionally implicate VEGF-C as a putative therapeutic target in GBM, thereby extending its function beyond the blood and lymph vessel formation.

**Figure 1:**
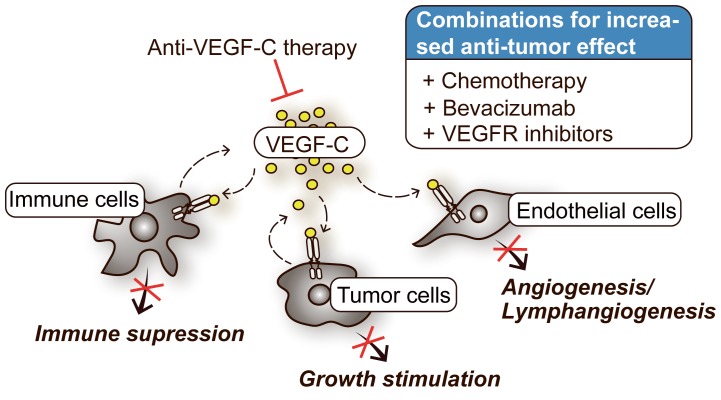
The role of VEGF-C signaling in cancer therapy.

The mechanisms underlying the upregulation of VEGF-C in cancer are poorly understood [3]. We found that the expression pattern for VEGF-C in GBM patient tissue is highly heterogenous, both at inter- and intra-patient level. This implies that if used as a predictive biomarker, such expression pattern would indicate VEGF-C targeting as a feasible therapeutic approach for only a subset of GBM patients. Besides its vessel stimulating effect, paracrine effects of VEGF-C signaling in cancer have mostly been linked to its immunosuppressive effect on natural killer cells, dendrite cells and T cells [3]. Since VEGF-C is secreted by both stromal and GBM cells [4, 5], one should take into consideration that it may also stimulate VEGFR-positive tumor cells that do no express VEGF-C in a paracrine manner. Due to this dual mode of action, even patients with cancer cells negative for VEGF-C and/or VEGFR2 expression may benefit from VEGF-C inhibition by impeding its paracrine functions. The heterogenous expression pattern of VEGF-C also suggest that a VEGF-C therapeutic approach may be more efficient in combination with other treatment modalities rather than as a monotherapy (Figure 1). Results from gastric cancer studies have shown an increased sensitivity to chemotherapeutic agent cisplatin when VEGF-C was silenced [6], thereby supporting the superiority of the combinational approach. Our data demonstrated that the activation of DNA damage response pathway increases upon VEGF-C silencing, indicating a novel role for VEGF-C as a sensitizer to DNA damaging therapies. In GBM, the humanized anti-VEGF-A antibody bevacizumab was FDA-approved for the treatment of recurrent GBMs already in 2009. However, therapeutic effect of bevacizumab has been questioned following the publication of placebo-controlled clinical phase III trials conducted in both the first-line and recurrent setting, due to its non-significant impact on the overall patient survival [7]. Our observations indicate a constitutive VEGF-C expression and activation of VEGF-C/VEGFR2 signaling under bevacizumab treatment both *in vitro* (exposure of primary GBM cells) and *in vivo* (analysis of matched GBM tumors sections prior and after bevacizumab treatment). This indicates the contribution of VEGF-C to bevacizumab resistance and stresses the therapeutic potential of combining bevacizumab with anti-VEGF-C therapy. A study by Dufies *et al*. reported increased VEGF-C expression as an acquired resistance mechanism under treatment with pan-kinase targeting inhibitors of VEGFRs; sunitinib, axitinib, pazotinib and sorafenib [8]. Using renal cell carcinoma cells, they claimed the mechanism underlying sunitinib stimulated VEGF-C increase involves both enhanced transcriptional activity and stabilization of VEGF-C mRNA. This study further supports the putative benefit of combined VEGF-C inhibition with pan-kinase inhibitors like sunitinib. Such therapeutic strategy may also proove relevant for GBM, where VEGFR inhibitors such as sunitinib failed to show a clinical benefit as monotherapy [7]. An anti-VEGF-C specific humanized antibody, VGX-100, has been interrogated in preclinical setting using acute myloid leukemia cells and proven to suppress the *in vivo* progression by inducing cellular differentiation [9]. An early clinical phase I evaluation in cancer patients showed that this treatment is well tolerated with pontential anti-tumor effect [10].

Alltogether, the data reported by us and others raise an important issue regarding VEGF-C as a compensary mechanism for various treatments and shows the feasibility of anti VEGF-C therapy as part of the therapeutic intervation. However, the translational aspect of VEGF-C targeting in patient treatment requires a better understanding of these compesatory responses inherent to cancer cells and will remain an intensive area of research for many malignancies not just the therapeutically resistant GBM.
